# Adult autologous mesenchymal stem cells for the treatment of suspected non-infectious inflammatory diseases of the canine central nervous system: safety, feasibility and preliminary clinical findings

**DOI:** 10.1186/s12974-015-0402-9

**Published:** 2015-09-29

**Authors:** Offer Zeira, Nimrod Asiag, Marina Aralla, Erica Ghezzi, Letizia Pettinari, Laura Martinelli, Daniele Zahirpour, Maria Pia Dumas, Davide Lupi, Simone Scaccia, Martin Konar, Carlo Cantile

**Affiliations:** San Michele Veterinary Hospital, Via Primo Maggio 37, 26838 Tavazzano con Villavesco, Italy; Department of Veterinary Sciences, University of Pisa, Viale delle Piagge 2, 56124 Pisa, Italy

**Keywords:** Central nervous system, Meningoencephalitis, Mesenchymal stem cells, Dog

## Abstract

**Background:**

Non-infectious inflammatory diseases of the canine central nervous system (CNS) are common idiopathic disorders grouped under the term meningoencephalomyelitis of unknown origin (MUO). Ante mortem diagnosis is achieved via assessment of clinical signs, magnetic resonance imaging (MRI), and cerebrospinal fluid (CSF) analysis, but the definitive diagnosis needs histopathological examination. MUO are mostly considered as autoimmune CNS disorders, so that suppressing the immune reaction is the best management method for patients. Mesenchymal stem cells (MSCs) are under investigation to treat autoimmune and degenerative disorders due to their immunomodulatory and regenerative properties. This study aims to verify the safety, feasibility, and efficacy of MSCs treatment in canine idiopathic autoimmune inflammatory disorders of the CNS.

**Methods:**

Eight dogs presented with acute onset and rapid progression of multifocal neurological signs were selected to the study. In all patients’ physical and neurological examinations, MRI and CSF analyses were performed. Clinical diagnosis in all cases was MUO. All selected dogs responded initially to immunosuppressive drugs (prednisone and a combination of prednisolone and cytosine arabinoside) but developed undesirable side effects. For all eight dogs, the owners considered euthanasia but accepted cell therapy as a last possibility. Autologous bone marrow MSCs (BMMSCs), isolated, cultured, and expanded, were administered by intrathecal (IT) injection in the cisterna magna intravenously (IV) and by intra-arterial (IA) injection in the right carotid artery. Adverse effects and clinical response were monitored for 6 months up to 2-year follow-up.

**Results:**

The use of autologous BMMSCs in dogs with MUO was safe for IT, IV, and IA injections. No major short- or long-term adverse effects were registered. All the dogs presented early improvement in their general and neurological conditions, with particular effect on cervical pain. The group of dogs treated by IT+IA administration showed a shorter time of reaction to therapy compared to the group treated by IT+IV administration.

**Conclusions:**

MSCs treatment in dogs affected by MOU is safe and feasible. A larger group of dogs is needed to confirm these results as well as CNS histology in order to better understand the underlying mechanisms.

## Background

The canine non-infectious inflammatory diseases of the central nervous system (CNS) are common diseases that can affect the brain, spinal cord, and/or the meninges. Clinical signs of non-infectious CNS inflammatory disorders are frequently very similar to those of infectious CNS diseases and even those of neoplasia [1]. The major diagnostic decision is between infectious and non-infectious diseases. Recently, the term meningoencephalitis of unknown origin (MUO) has been introduced to all clinically diagnosed cases based on advanced imaging and cerebrospinal fluid (CSF) analysis of non-infectious inflammatory CNS disease [1–3]. MUO includes all the specific subtypes of non-infectious inflammatory disease that can be identified through histopathology, including granulomatous meningoencephalomyelitis (GME) and necrotizing encephalitis (NE), but does not include the diseases without evidence of explicit CNS involvement, such as steroid-responsive meningitis-arteritis (SRMA) [1]. The inclusive term NE incorporates necrotizing meningoencephalitis (NME) and necrotizing leukoencephalitis (NLE) because of the overlapping in clinical signs, signalment, and neuropathology. They differentiate on the region of the brain involved [1, 2]. Particularly, NME is an idiopathic inflammatory disease of the CNS that is characterized by prominent necrosis and infiltration of inflammatory cells, including lymphocytes, plasma cells, and monocytes or histiocytes into the cerebral cortex and/or white matter, hippocampus, thalamus, and leptomeninges. NME has been reported in various canine breeds, including the Pug [4, 5], Maltese [6, 7], Shih tzu [8], Papillon [8], Chihuahua [9], Pekingese [10], Yorkshire terrier [11, 12], and French bulldog [13]. The areas of necrosis and inflammatory cell infiltration are localized in the cerebral cortex and subcortical region in most NME cases, whereas in a few breeds, such as the Yorkshire terrier and French bulldog, the lesions are predominately observed in the white matter, and the disease is called NLE.

GME represents up to 25 % of all canine inflammatory CNS diseases. Neurological signs are non-specific and can be localized to the forebrain, brainstem, or spinal cord or appear as a multifocal syndrome [1, 14]. The clinical presentation correlates with three pathological distributions: multifocal or disseminated (clinically characterized by acute onset and rapid progression of neurological signs, fever, paraspinal hyperesthesia, mainly localized to the cervical region); focal (slower progression of neurological signs, the differential diagnosis including intracranial neoplasia); or ocular (optic neuritis, anterior, and posterior uveitis). GME’s neuropathological lesions consist of perivascular cuffs composed of lymphocytes, plasma cells, macrophages, and some neutrophils, as well as granulomatous lesions containing epithelioid cells, mainly in the cerebellum and brainstem [15].

The etiopathogeneses of GME and NE remain unclear. MUO has long been assumed to have an autoimmune and genetic pathogenesis. Autoimmune diseases arise from dysregulation of either or both of the innate and adaptive immune systems to produce inflammatory responses leading to cellular dysfunction and tissue destruction. In general, major factors that contribute to the development of autoimmunity are genetic susceptibility and environmental factors (e.g., infections, tissue injury). Nevertheless, a trigger factor is assumed to initiate signs of disease in each specific dog at a specific time [15, 16]. Suspected agents include environmental or infectious antigenic triggers that might activate autoreactive cells in the CNS, although no such agent has yet been incriminated in the development of MUO [17, 18]. Susceptibility genes may confer susceptibility or protection for autoimmunity by influencing the maintenance of self-tolerance. One of the most important features of the CNS is its relative isolation from the peripheral immune system, which has important implications regarding the pathogenesis, diagnostic criteria, and therapy for inflammatory CNS diseases [19]. The blood-brain barrier (BBB) and the blood-spinal cord barrier implies that there is “gating” of the flow of cells and macromolecules from the systemic circulation to the CNS [20] and that CNS is immunocompetent and actively interacts with the peripheral immune system [21]. Although neuroinflammation has been investigated in several spontaneous canine CNS diseases, mechanisms still remain enigmatic for the MUOs. Immunomodulation through cross-talk between the periphery (extraneural) and the CNS, and how to limit cytotoxicity and enhance neuroprotection, would help identify appropriate targets for immune-based therapy.

Administration of immunosuppressive doses of glucocorticosteroids is the traditional primary treatment in MUO in dogs which may help reduce inflammatory and immune reactions during initial stage of the disease [22]. This results in remission of clinical signs for a period of time, but many dogs require sustained therapy to avoid relapse. Prognosis is poor and long-term therapy causes many complications [22, 23]. Adverse effects such as polyuria-polydipsia, polyphagia, weight gain, hepatotoxicity, iatrogenic hyperadrenocorticism, and lethargy are frequently seen during long-term therapy. Thus, alternative treatments for MUO are required due to these disadvantages [22, 23].

Reported second-line immunosuppressive drug therapies used for the treatment of MUO cases are procarbazine, cytosine arabinoside, lomustine, azathioprine, and cyclosporine [1, 22, 23].

Many of these drugs have potential risks for myelosuppression, hepatotoxicity, and gastrointestinal disturbance.

Convincing immunomodulatory features have been ascribed to MSCs derived from the bone marrow, an easily accessible and highly proliferative stem cell source [24–27]. While the prominent benefit provided by these multipotent adult stem cells has been largely documented in several disease models, being characterized by inflammatory reactions in the nervous system (such as amyotrophic lateral sclerosis (ALS), Parkinson’s disease, Alzheimer’s disease, or traumatic injuries), it is noteworthy that the immunomodulatory capacity of grafted MSCs does not necessarily depend on cell-specific differentiation or the integration of the grafted cells into the host tissue. It rather seems that MSCs possess the potential to establish a transient neurotrophic microenvironment that is beneficial and supports tissue healing, repair, and regeneration. In fact, grafted MSCs have been recently described as “in vivo drugstores,” synthesizing and secreting paracrine factors, which mediate therapeutic benefits [28–31]. Supporting this concept, two in vitro studies have documented that MSCs can release growth/neurotrophic factors as well as anti-inflammatory proteins and modulate microglial responses to pro-inflammatory stimuli [32, 33]. Moreover, single intra-brain or intravenous injections were shown to ameliorate neuroinflammation and associated behavior in animal models of neuropathic pain [34, 35].

In the present investigation, we evaluate the safety, the feasibility, and the efficacy of MSC treatment in canine idiopathic autoimmune inflammatory disorders of the CNS.

## Materials and methods

### Dog population

MUO is a clinical diagnosis based on neurologic examination, cross-sectional imaging findings, and CSF abnormalities, supplemented by exclusion of infectious diseases; definitive diagnosis requires histopathology. In our study, we followed the guidelines proposed by Granger and colleagues [14] in order to establish an ante mortem presumptive diagnosis of MUO in the absence of histopathologic diagnosis.

The study population consisted of eight dogs, four males and four females, between 1 and 4 years old, referred to the San Michele Veterinary Hospital, Tavazzano con Villavesco, Lodi, Italy, between 2009 and 2014. All dogs were suspected of non-infectious inflammatory disease of CNS. The clinical diagnosis was supported by signalment, history, neurologic examinations, complete blood count, serum biochemistry profile, and cisterna magna CSF collection.

CSF analysis consisted in Burker chamber cell counting, cytological evaluation of an air-fixed, May-Grünwald Giemsa-stained sediment and protein concentration.

Polymerase chain reaction (PCR) analysis performed for toxoplasmosis, neosporosis, canine distemper, ehrlichiosis, and leishmaniasis was negative. MRI has been performed using a 0.25T magnet (Esaote Vet MR Grande). Imaging protocol consisted of eight sequences: transverse and sagittal T2W FSE, dorsal fluid attenuated inversion recovery (FLAIR), dorsal Hyce 3D, T1W transverse SE, and T1W dorsal Turbo 3D. In all dogs, the last two sequences were repeated after the intravenous administration of 0.3 ml/Kg BW contrast medium (Omniscan GE Healthcare).

In order to perform MRI and CSF collection dogs were sedated with medetomidine (5 μg/kg) and buprenorphine (5 μg/kg) by intramuscular injection; after 20 min, they were induced to general anesthesia with diazepam (0.25 mg/kg) and propofol (4 mg/kg) then intubated with an oro-tracheal catheter and maintained with isoflurane (MAC 1.1). In order to be included in the study, each dog needed to present neurological signs, be positive to at least one out of the two parameters: MRI or CSF and negative to PCR exams for the various infectious diseases. Finally, in order to be eligible, all candidates underwent thorax radiographs and abdominal ultrasound, so that other diseases could be ruled out.

### Treatment

Selection of a specific therapy protocol depended on the clinician’s decision, the patient’s clinical status, and the pet owner’s financial and personal considerations. Generally, all suspected MUO cases are treated in our hospital initially by immunosuppressive doses of prednisone (2 mg/kg/day) and tapered with response over the following months to achieve the lowest dose possible that controls signs. When necessary, the therapy is supported by cytosine arabinoside administered at 50 mg/m^2^ every 12 h as a subcutaneous bolus for two consecutive days once every 3 to 4 weeks for 3 cycles and then increasing progressively the interval between treatment cycles. If a relapse occurred, the protocol is repeated with the initial doses.

From all our MUO cases, eight dogs were selected for two main reasons: first, they all relapsed after prednisone was stopped and responded poorly to a second full dose therapy, and second, the owners asked for euthanasia but accepted cell therapy as a last possibility.

Three of the dogs were treated by a combination of prednisone and cytosine arabinoside administered as described above but developed undesirable side effects. Three dogs presented seizures, one of them was treated with phenobarbital (PB), 3 mg/kg twice a day, and the other two also with levatiracetam, 10 mg/kg every 8 h as anticonvulsant therapy.

### Ethics statement

All animal procedures involving MSCs were performed in accordance with the guidelines defined by the Italian Presidency of the Council of Ministers and following the guidelines published by the General Directory of Animal Health and Veterinary drugs of the Italian Ministry of Health [36].

The owners of the eight dogs recruited for the MSC treatment were thoroughly informed about the entire procedure and signed a formal agreement with the San Michele Veterinary Hospital in acceptance of the therapy. Owners have also accepted that their dogs will undergo post-mortem examination at the end of their life.

### BMMSCs collection, isolation, culture, and quality controls

Dogs were sedated and anesthetized, as previously described, and with the animals in lateral recumbency, autologous bone marrow (BM) was collected from the proximal end of both humeri using a T-handle Jamshidi® needle (14G for large dogs, 18G for small dogs).

Of BM, 40 ml was collected and diluted 1:4 with heparinized culture medium (Dulbecco’s modified Eagle’s medium (DMEM) with L-Glutamine, 4500 mg/L D-Glucose, 25 mM HEPES, without sodium pyruvate and supplemented with 10 % inactivated fetal bovine serum and 1 % penicillin-streptomycin (Gibco, Life Technologies) and 1 % Glutamax (Gibco, Life Technologies). BM was diluted again 1:4 with Dulbecco’s phosphate buffered saline (DPBS without calcium, magnesium—Gibco, Life Technologies), and peripheral mononuclear cells (PBMCs) were isolated using LeucoSep^TM^ tubes preloaded with Ficoll (Greiner Bio One). The PBMCs was harvested by a pipette, inserted in an individual tube, and washed by centrifugation in DPBS twice. The resultant cell precipitate was suspended in culture medium supplemented with EGF (Sigma) and bFGF (Gibco, Life Technologies). The cell suspension was transferred to T25 flask (Greiner Bio One) and incubated at 37.5 °C and 5 % humidified CO_2_. The medium with non-adherent cells was discarded after 24 and 48 h, the cultures were carefully washed in DPBS with calcium and magnesium (Gibco, Life Technologies), and culture medium was replaced with a fresh portion. The medium was then replaced every 2–3 days. After attaining a sub confluent state, the cells were removed with 0.05 % trypsin-EDTA (Gibco, Life Technologies) and seeded again into new flasks at split ratio of 1:3. Cultures were expanded for only three passages. MSCs were characterized according to morphology, growth traits, and cell-surface antigens profile (fluorescence-activated cell sorting (FACS)). For FACS analysis 1 × 10^6^ cells were resuspended in each flow cytometry tubes containing 500 ml of DMEM with 10 % FBS and were labeled using the following directly conjugated antibodies: CD45-fluorescein isotyocianate (-fitc) (clone YKIX716.13, AbD Serotec), CD14-pycoerythrin (-pe) (clone TUK4, AbD Serotec), CD34-pe (clone 1H6, Pharmingen), CD117-pe (clone ACK45, Pharmingen), and CD44-fitc (clone IM7, Pharmingen). After 20-min incubation at 4 °C, tubes were washed twice in PBS and acquired using a FacsCalibur (Becton Dickinson) flow cytometer. Cells were identified as a population of large and moderately complex cells based on FSC vs SSC scattergram, and positivities were expressed in percentage in comparison with appropriate isotypic controls. Bacterial and fungi contaminations were tested with microbiological analysis, and mycoplasma contamination was excluded with DAPI stain. For DAPI, 500 ul of cell suspension at the third culture passage were plated in a Petri culture dish and incubated for 48 h. The cells were fixed with 4 % paraformaldehyde with 2 % sucrose in DPBS, post-fixed with 70 % ethanol, and put at −20 °C for at least 1 h. The cells were incubated with DAPI (Sigma) 1:100000 for 15 min and examined and photographed under a fluorescence microscope.

### BMMSCs administration

Bone marrow MSCs (BMMSCs) at passage three (approximately 14 days of growth) were used for the therapy. The dogs were sedated, induced to general anesthesia, as previously described, and received their autologous BMMSCs diluted in saline solution. Three dogs received 2 × 10^6^ cells intrathecally (IT) in cisterna magna and 4 × 10^6^ cells by injection in the right carotid artery after it has been surgically exposed as routinely done for the intra-arterial (IA) administration of drugs [37].

Four dogs received 2 × 10^6^ cells IT in the cisterna magna and 0.5 × 10^6^ cells/kg intravenously (IV). The last dog, that was treated twice, had an IT+IV administrations the first time and IT+IA the second time (13 months after the first one) (Table 2).

### Follow-up

Eventual adverse effects and clinical outcomes were monitored up to 2 years and a minimum of 6 months, in one case, after treatment with BMMSCs. Follow-up included neurological examinations, urinalysis, complete blood, and biochemistry work-up 3 months after treatment. A control MRI and neurologic assessment were done 6 months after treatment. If necessary, dogs who had abnormal results, repeated the specific exams 1–3 months later. During the whole 24 months, owners and referring veterinarian forwarded their surveys on a monthly base or whenever they considered it necessary.

### Histopathology

The brain and spinal cord and samples of major organs were fixed in phosphate buffered 4 % formalin solution. Tissue samples were routinely processed for histology, and sections were stained with hematoxylin and eosin, luxol fast blue, periodic acid-Schiff, Grocott’s methenamine silver, and Gram stainings.

## Results

### BMMSCs culture

The cultured MSCs consistently (>98 %) expressed their classical surface markers and were negative for lymphocytes and hematopoietic cells (FACS analysis) and for bacteria and fungal and mycoplasma contaminations. Cultures were ready for administration within a maximum period of 14 days. In four cases, the cell quantity obtained permitted cryoconservation of unused cells.

### General and neurological status

At the time of presentation, neurologic examination revealed non-specific signs, localized to forebrain, brainstem, cerebellum, or spinal cord, or appeared as a multifocal syndrome. In most cases, clinical signs consisted of an acute onset of cervical pain, tremors, ataxia, paresis, and altered mental status and, in three cases, seizures. A progressive deterioration of the clinical status of all dogs was reported. The results of the collected clinical data are presented in Table 1.

**Table 1:** Dog population, initial neurologic signs, and clinical diagnosis work-up

Case	Breed	Sex	Age (years)	Neurologic signs	MRI	CSF
1	Labrador retriever	M	4	Acute onset of tremors, severe ataxia, cervical pain	Single brainstem lesion, hyperintense in T2 and FLAIR, variable contrast enhancement	Mononuclear pleocytosis
2	Chihuahua	M	4	Acute onset of depression, compulsive walking, cervical pain	Normal	Mononuclear pleocytosis
3	Half-breed	M	3	Acute onset of general tremors, tetraparesis, cervical pain	Diffuse spinal cord lesion at C3-C4, hyperintense in T2 and FLAIR, severe contrast enhancement	Mononuclear pleocytosis
4	Golden retriever	F	2	Acute onset of compulsive walking, cervical pain, depression, tetraparesis	Normal	Mononuclear pleocytosis
5	Cocker Spaniel	F	3.5	Peracute onset of circling, ataxia, vision deficit, cervical pain	Multifocal lesions in both white and gray matter, hyperintense in T2 and FLAIR, no contrast enhancement	Mononuclear pleocytosis
6	Poodle	F	1	Acute onset of severe circling, head tilt, seizures	Multifocal cerebrum and brainstem lesions, hyperintense in T2 and FLAIR, no contrast enhancement	Mixed pleocytosis (lymphocytic/mononuclear)
7	Yorkshire terrier	M	1.5	Acute onset of alternate mental status agitation-stupor, seizures	Diffused hyperintensity in T2 an FLAIR in all left brain hemisphere, variable contrast enhancement	Mononuclear pleocytosis
8	Boxer	F	2	Peracute onset of severe cervical pain and weakness, tremors, partial seizures	Multifocal lesions hyperintense in T2 and FLAIR, variable contrast enhancement	Mixed pleocytosis (mainly mononuclear)

During the BMMSCs culture period, all dogs continued prednisolone therapy and seven dogs used tramadol at 3 mg/kg/tid, due to paraspinal cervical pain. Owners reported only mild effects of this therapy.

All dogs had normal blood work-up and urinalysis through the follow-up period. No adverse effects followed BMMSCs administration at any time, except for one case in which a transitory hyperthermia was registered 24 h after BMMSCs delivery. Owners and referring veterinarians reported an initial improvement in general and neurologic status within 5 days after treatment in the IT+IA group of dog and 13 days after treatment in the IT+IV group. In particular, paraspinal hyperesthesia of the cervical region, which was a major feature in all dogs, improved. This has been confirmed in the first neurologic control 30 days after treatment, together with improvement of behavior changes, depression, proprioception and postural reactions, ataxia, head tilt, and circling. After the first neurologic control, improvements were variously progressive through a maximum period of 6 months and were not registered afterward. At 6 months, four dogs were completely normal, one dog maintained mild ataxia as a single clinical sign, two dogs needed to continue anti-epileptic treatment but were otherwise normal, and one dog died for reasons not related to its neurologic disease.

After BMMSCs treatment, the two dogs under anti-epileptic therapy presented reduction in seizures frequency and one of them also in seizure intensity.

**Fig. 1:**
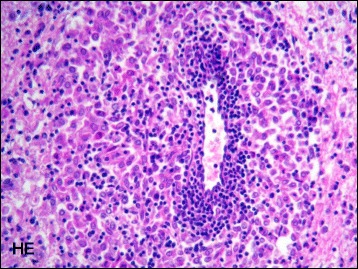
Histopathologic examination evidenced an angiocentric inflammatory lesion in the cerebral white matter composed of macrophages and mixed with lymphocytes and plasma cells (hematoxylin and eosin, ×400)

The dog who died had a definitive post-mortem diagnosis of GME (Fig. 1).

### CSF and MRI

All dogs underwent control CSF (3 months after treatment) and control MRI (6 months after treatment).

**Fig. 2:**
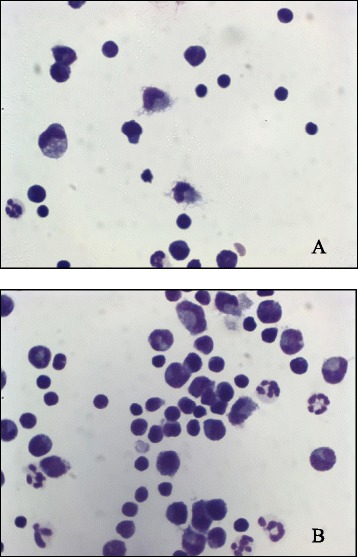
Cytological preparation of the cerebrospinal fluid of cases 2 (**a**) and 4 (**b**). **a**, **b** mononuclear pleocytosis. The cellular population was mainly represented by monocytes, macrophages, and lymphocytes. A small number of non degenerated neutrophils were also present (May-Grunwald Giemsa stain, ×100)

**Fig. 3:**
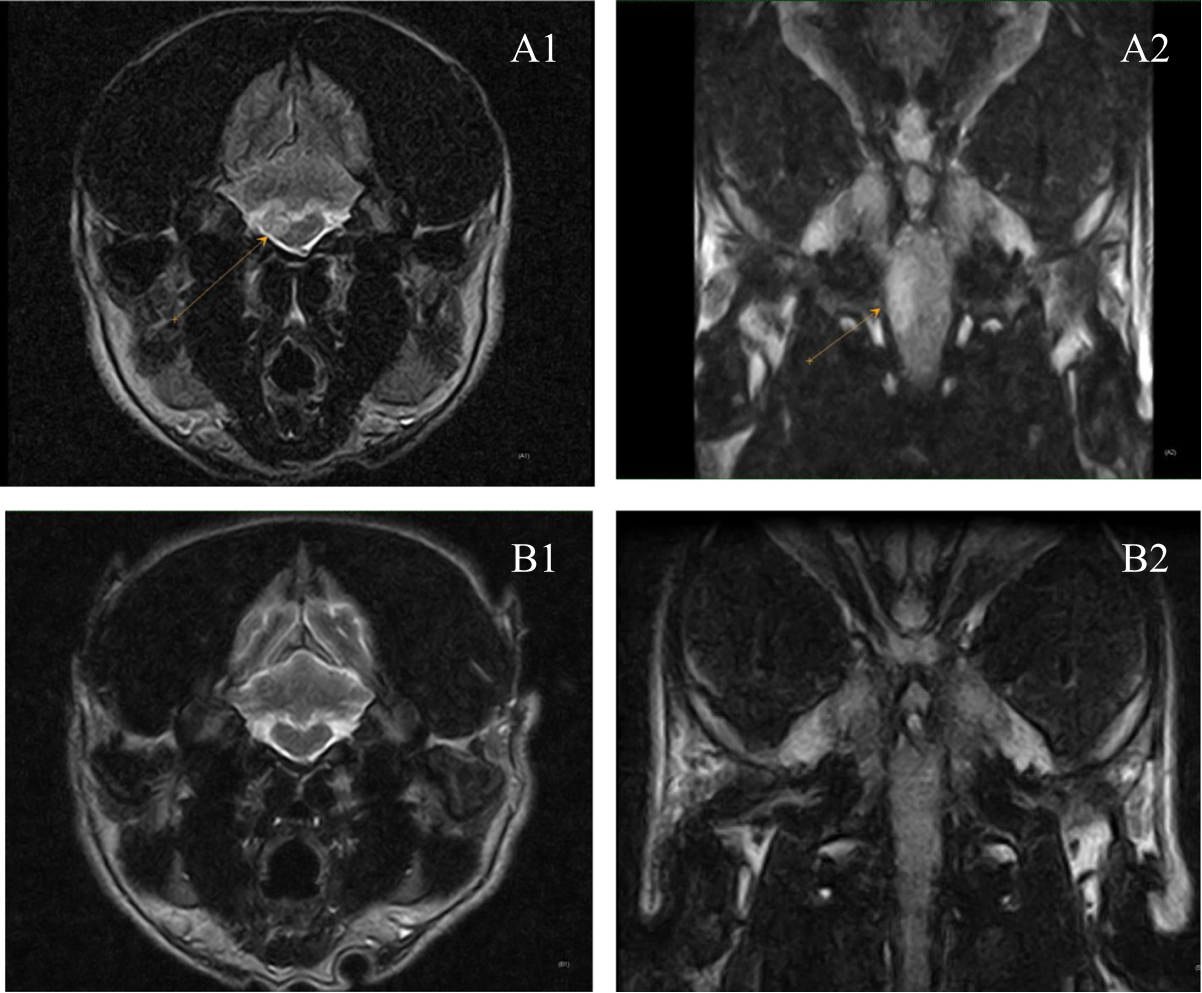
Dorsal and transversal MRI images of brainstem MOU lesion before and 6 months after treatment. The first MRI exam (*A1* and *A2*) showed a single intra-axial hyperintense lesion (*arrow*) on the right side of brain stem, not too well-delineated, visible in T2-weighted sequences (*A1*) and FLAIR sequences (*A2*). There was no evidence of the lesion in the control MRI exam (*B1* and *B2*) performed 6 months later

**Fig. 4:**
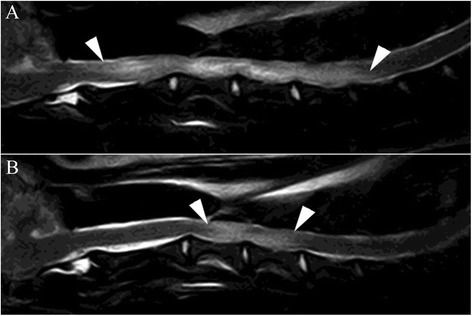
Sagittal T2 (FSE T2, TR 4710 TE 120; 3-mm slice thickness) through the cervical spine. **a** Before and **b** 40 days after initiation of therapy. **a** shows increased intramedullary signal from mid of C2 until mid of C6 (*arrowheads*). The changes are consistent with inflammatory edema. **b** The visible extent of the inflammation is significantly decreased—changes reach now from mid of C3 to the end of C4 (*arrowheads*)

At the time of presentation, CSF showed mixed pleocytosis with >50 % mononuclear (monocytes/lymphocytes) cells, some neutrophils, and increased protein concentration (>30 mg/dl) (Fig. 2). Within the selected group of dogs, six patients showed multiple, single, or diffuse intra-axial hyperintense lesions on T2W and FLAIR images, variable T1W contrast enhancement, gray and white matter lesions, and variable mass effect, while two dogs had a negative MRI with positive CSF. Three months after treatment, one dog still presented a mononuclear pleocytosis (>8 cells/μl, represented by monocytes and lymphocytes) but had normal CSF 6 months after treatment (Table 2). Six months after treatment, five dogs had normal MRI; in two dogs, the lesions initially detected appeared better, and in one, MRI lesions were unchanged (Figs. 3 and 4). Two of these dogs underwent MRI 12 and 24 months after treatment and, at that time, were negative to the lesions initially detected. All dogs remained free of any therapy through the follow-up period, except for the two dogs under anti-epileptic drug, and one dog that had repeated MSC treatment after 1 year due to reappearance of mild ataxia. Seven dogs of the group are alive at present time (Table 2).

**Table 2:** Initial treatment, BMMSC treatment, and follow-up

Case	Initial treatment	MSCS treatment (IT/IA/IV)	3 months (CSF)	6 months (MRI/CSF)	12 months (MRI/CSF)	24 months (MRI/CSF)
1	Prednisone	IT+IA	Negative	MRI negative		
2	Prednisone	IT+IA	Positive	MRI negative CSF negative		
3	Prednisone Cytarabine	IT+IA	Negative	MRI better CSF negative		
4	Prednisone	IT+IV after 1 year also IT+IA	Negative	MRI negative	MRI negative CSF negative	MRI negative CSF negative
5	Prednisone Cytarabine	IT+IV	Negative	MRI better	MRI unchanged CSF negative	MRI unchanged CSF negative
6	Prednisone, PB, levetiracetam	IT+IV	Negative	MRI unchanged		
7	Prednisone, PB	IT+IV	Negative	MRI negative		
8	Prednisone, PB, levetiracetam, cytarabine	IT+IV	Negative	MRI negative

### Histopathology

Post-mortem histological examination revealed lesions limited to the CNS, characterized by dense aggregates of inflammatory cells arranged in perivascular cuffs. Lesions mainly involved the white matter of the telencephalon, caudal brain stem, cerebellum, and cervical spinal cord. Involvement of meninges related to lesions of white matter directly underlying was also observed. The inflammatory cell aggregates were composed principally of macrophages admixed with lymphocytes and plasma cells. Frequently, a focal eccentric accumulation of macrophages within the perivascular cuffs was evident (Fig. 1). In the most severely affected areas, the adjacent nervous tissue was edematous and a diffuse dissemination of mononuclear cells was observed. No bacteria, protozoa, or fungi were detected by the histochemical stainings. The morphological pattern of the inflammatory lesions was consistent with a disseminated form of canine granulomatous meningoencephalomyelitis (GME).

## Discussion

The field of immune modulation is expanding rapidly in the last few years in both human and veterinary medicines. Many autoimmune diseases of the nervous system in human medicine have been identified in veterinary medicine. A classic example of autoantibody-mediated disease is myasthenia gravis; in this disease, autoantibodies have been shown to target the muscle acetylcholine receptor. Another example is the Guillain-Barré syndrome (GBS), where strong evidence supports an important role for antibodies to gangliosides [38]. Analog diseases exist in veterinary medicine. In human medicine, it is now well-established that a substantial proportion of these diseases are associated with autoantibodies directed against the extracellular domains of cell-surface proteins which are critical in the regulation of neuronal excitability. There are conclusive clinical and scientific data to support the pathogenicity of the antibodies including the correlation between antibody levels and severity of clinical features in an individual and the development of similar diseases in experimental animals after antibody transfer [39]. Furthermore, serum levels of some antibodies are usually higher than CSF levels. Probably, the disease begins in the periphery and not in the brain; however, upon relating to total IgG concentrations, this ratio is reversed showing “intrathecal synthesis” [40–42].

In human medicine, improvements were greatest in the patients with autoimmune encephalitis after early administration of steroid therapies [39]. Neurologists are now frequently recognizing this condition as an immunotherapy-responsive encephalopathy.

In veterinary medicine, neuroinflammation has been investigated in several spontaneous canine CNS diseases. Although the precise mechanism in MUO remains unclear, some findings suggest similarity to the mechanisms described in human autoimmune encephalitis [43–47]. While in human medicine, various mouse experimental autoimmune encephalitis were treated by cell therapy [48], to the best of our knowledge, MSCs were never used in similar pathologies in dogs. In the light of these facts, and since the spontaneous dog’s model has been accepted for the research on some human neuromuscular diseases, it is interesting to analyze the safety and efficacy of MSCs treatment in the dog’s spontaneous MUOs. This may open new therapeutic possibilities in dogs that do not respond to standard therapy or those in which standard therapy is impossible for different reasons. Furthermore, if proved safe and efficient, this treatment may contribute to create protocols for human treatment of similar diseases.

In our study, the use of standardized laboratory procedures and protocols permitted to produce, for each dog, the adequate therapeutic BMMSC dose allowing us to guarantee a comparable result. Furthermore, shortening culture time to 12–14 days was of special benefit to the dogs that presented the more severe clinical signs. Obtaining major cell quantity permitted cryoconservation of unused cells for an eventual future treatment. Alongside the benefit to the patient, we noted that this possibility helped the owners of the dogs to decide for BMMSC treatment rather than euthanasia.

In our group of dogs, we could not find any short- or long-term major adverse effect to the BMMSC therapy. In the short term, the only dog, in which a transitory hyperthermia was registered 24 h after treatment, had no other clinical signs. Such a reaction, if indeed related to the treatment, may be considered as a minor adverse reaction. In the longest term, two dogs who underwent MRI 12 and 24 months after BMMSCs treatment were both free of any pathology visible by this method.

The use of autologous BMMSCs from the bone marrow in dogs with MUO was feasible. Intrathecal injection is a routine procedure that can be done by any neurologist, while intra-arterial injection should be considered as a minor surgical procedure, since exposure of the carotid artery is necessary; therefore, the participation of a surgeon is needed.

Early clinical improvement has been reported by owners and referring veterinarians. Both reported improvement in the dog’s general conditions. In particular, paraspinal hyperesthesia of the cervical region, which was a major feature in all dogs, disappeared. This has been confirmed in the first neurologic control 30 days after treatment, together with improvement of the initial behavior changes, depression, proprioception and postural reactions, ataxia, head tilt, and circling. Interestingly, in seven dogs in which tramadol was used due to paraspinal cervical pain during the waiting period for treatment, owners reported only mild effects of this therapy compared to BMMSCs’ effect. This result is in line with previous reports of amelioration of neuroinflammation and associated behavior in animal models of neuropathic pain after a single intra-brain or IV injection of BMMSCs [35, 36].

Although theoretically, IV injection may be sufficient and equally effective because BMMSCs exert peripheral immunomodulation effects, we believe that IT- and IA-injected BMMSCs, besides their local anti-inflammatory and immunomodulation effects, may circulate with the arterial blood flow and CSF having better possibility to reach the affected areas in the CNS, inducing a superior neurotrophic and neuroprotective effects.

Analyzing IT+IV compared to IT+IA administration shows that they achieved similar response but in different times. The group of dogs treated by IT+IA administration showed a shorter time of reaction to therapy compared to the group treated by IT+IV administration. Even though IT+IA group was up to 8 days faster in improving the clinical status, within 2 weeks, no difference was registered in the clinical course of both groups. The only dog treated twice also showed a more rapid response to the IT+IA administration compared to the IT+IV injections. This may be explained by the faster distribution of BMMSCs via the carotid artery.

Another interesting aspect is that recently, in human medicine, it has become clear that a number of patients with encephalitis associated with some antibodies complexes have a specific seizure semiology. These patients showed an excellent response to corticosteroids, often administered after multiple anti-epileptic agents were ineffective [49]. This response was paralleled by a decrease in the antibody complex levels. The concept is particularly well-demonstrated in patients with frequent seizures whose seizure frequency is often dramatically reduced with corticosteroids [49–51]. Three of the eight dogs affected by MUO had seizures; in one case, they were acceptably controlled with PB, with frequency of once every 2 months. Nevertheless, other two dogs that were treated with a combination of PB and levatiracetam were unstable; one had three to four seizures a month and the other one to two seizures a month.

Six months after they initiated the BMMSCs therapy, both dogs had an average of one to two seizures every 45 days, and one presented a lower seizure intensity, which contributed to the quality of life of both dogs and owners. It may be possible that in these cases, seizures were, at least partially, dependent on autoimmune mechanisms.

Finally, as far as the long-term survival, it is worthwhile underline that all but one dog are alive at the present time; they were treated from 1 to 4 years ago, with overall median survival of 705 days. All the patients conduct normal life; their veterinarians report normal clinical status, except for the anticonvulsant therapy in two cases, and the owners reported to be satisfied with their initial decision to try the therapy. Literature and various authors report an overall median survival, for dogs treated with corticosteroids and second-line immunosuppressive protocol, of 240 to 590 days, while dogs treated with corticosteroids alone or with lomustine survive from 28 to 357 days.

Moreover, dogs often respond initially to corticosteroids, but relapses are common; sustaining remission thus may require long-term high-dose corticosteroids or administration of alternative immunosuppressive agents whereby the undesirable side effects.

## Conclusions

The use of autologous MSCs from the bone marrow in dogs with MUO was feasible for both IT and IA injections, even though the second should be considered as a minor surgical procedure. The dogs presented early improvement in their general and neurological conditions, with particular effect on cervical pain. The group of dogs treated by IT+IA administration showed a shorter time of reaction to therapy compared to the group treated by IT+IV administration. Six months after they initiated the BMSCs therapy, both dogs under anti-epileptic therapy has reduced the frequency and one of them also the intensity, of seizures. The results of this study suggest that BMMSC treatment in dogs affected by MUO is safe and feasible. Furthermore, the treatment proved to be effective in reducing cervical pain and neurologic deficits, probably due to anti-inflammatory and immunomodulation effects. A much larger group of dogs is needed to confirm these results as well as CNS histology in order to better understand the underlying mechanisms.

## Abbreviations

BBB: blood-brain barrier
BM: bone marrow
BMMSC: bone marrow mesenchymal stem cell
CNS: central nervous system
CSF: cerebrospinal fluid
GBS: Guillain-Barrè syndrome
GME: granulomatous meningoencephalomyelitis
IA: intra-arterial
IT: intrathecal
IV: intravenously
MRI: magnetic resonance imaging
MSC: mesenchymal stem cell
MUO: meningoencephalomyelitis of unknown origin
NE: necrotizing encephalitis
NLE: necrotizing leukoencephalitis
NME: necrotizing meningoencephalitis
PB: phenobarbital
PBMC: peripheral mononuclear cells
SRMA: steroid-responsive meningitis-arteritis
